# Clusterin activates the heat shock response via the PI3K/Akt pathway to protect cardiomyocytes from high-temperature-induced apoptosis

**DOI:** 10.1515/biol-2025-1082

**Published:** 2025-03-28

**Authors:** Jianguo Zhou, Xiupan Lu, Yiming Xie, Guangyao Mao

**Affiliations:** Department of Emergency, Taizhou People’s Hospital, Taizhou, 225300, Jiangsu, China; Department of Finance, Taizhou People’s Hospital, Taizhou, 225300, Jiangsu, China; Department of Central Laboratory, Taizhou People’s Hospital, No. 366, the Taihu Lake Road, Pharmaceutical High tech Zone, Taizhou, 225300, Jiangsu, China

**Keywords:** cardiomyocyte apoptosis, heat shock proteins, myocarditis therapy, oxidative stress

## Abstract

High temperature (HT) is a common symptom of infectious myocarditis. This study investigates the effects of HT on the heat shock response (HSR) and apoptosis in cardiomyocytes, with the aim of providing insights into potential treatment strategies for myocarditis. Rat cardiomyocytes (H9c2 cells) were exposed to 42°C for 1 h, followed by a return to 37°C to simulate high fever conditions. The cells were divided into seven groups: control, oe-NC, oe-CLU, HT, HT + oe-NC, HT + oe-CLU, and HT + oe-CLU + LY294002 (PI3K inhibitor). Protein levels of HSP70, HSP90, Bax, Bcl2, CLU, p-PI3K, and p-Akt were measured by Western blot, while mRNA expression of HSP70, HSP90, Bax, Bcl2, and CLU was assessed via reverse transcription quantitative polymerase chain reaction. Cell proliferation (cell counting kit-8 assay), apoptosis (flow cytometry), and reactive oxygen species (ROS) levels (MitoSOX assay) were also evaluated. HT exposure led to decreased cell proliferation, increased apoptosis, and elevated ROS levels (*p* < 0.001), while also inducing expression of HSP70 and HSP90 (*p* < 0.0001). Overexpression of Clusterin (CLU) enhanced HSP70 and HSP90 levels, reduced apoptosis, improved cell proliferation, and decreased ROS under HT conditions (*p* < 0.0001). The PI3K inhibitor reversed these protective effects, confirming the involvement of the PI3K/Akt pathway (*p* < 0.05). CLU activates the PI3K/Akt pathway, thereby enhancing the HSR and protecting cardiomyocytes. These findings suggest that CLU could be a potential therapeutic target for myocarditis treatment.

## 1 Introduction

Infective myocarditis (IM) is a common yet often underdiagnosed condition, resulting from the immune response to cardiac infection [1]. It is associated with the development of serious cardiac diseases, including dilated cardiomyopathy and chronic heart failure [2]. A comprehensive understanding of the pathophysiological changes in IM is essential for timely diagnosis and effective management of myocardial tissue damage in affected patients [3]. IM is frequently associated with high-temperature (HT) conditions [4], which can induce heat stress and trigger the heat shock response (HSR) [5]. HSR is a protective adaptive mechanism characterized by changes in gene expression in response to heat stress [6]. In various critical illnesses, including myocarditis, HSR plays a crucial role in mitigating acute injury [7]. Under heat stress, cells synthesize or upregulate a group of proteins known as heat shock proteins (HSPs). These HSPs are well-documented for their protective effects against myocardial ischemia–reperfusion injury and are increasingly recognized as potential therapeutic targets for cardiovascular diseases [8]. Additionally, HSPs have been implicated in the regulation of hypoxia/reoxygenation injury and apoptosis in cardiomyocytes [9]. However, it remains unclear whether HT can induce a robust HSR sufficient to counteract myocardial damage in IM. Further investigation into the molecular mechanisms by which HT activates HSR may open new avenues for therapeutic intervention in fever-induced myocardial injury.

Clusterin (CLU) is a stress-responsive, ATP-independent molecular chaperone that is upregulated in numerous diseases [10]. CLU plays a pivotal role in protein homeostasis, cellular apoptosis, survival signaling, and transcription regulation [11]. It has been shown to inhibit heat stress-induced apoptosis and exhibits chaperone-like activity similar to that of small HSPs [12], suggesting a close association with the HSR. However, whether HT modulates HSR by influencing CLU expression remains to be fully understood.

Cardiomyocyte injury is often linked to apoptosis, which is regulated by pathways involving proteins such as p53, NF-κB, and PI3K [13]. Numerous studies suggest a strong relationship between CLU and the PI3K/Akt pathway. For example, CLU has been shown to mitigate aging through activation of the PI3K/Akt pathway, prevent cell apoptosis via the PI3K/Akt/mTOR axis, and alleviate testicular injury [14]. Additionally, CLU promotes cellular survival and protects against oxidative stress through the PI3K/Akt pathway [15]. The PI3K/Akt pathway also regulates the expression of HSPs, including HSP27, which alleviates cardiac dysfunction, and HSP70, which is critical in cellular stress responses [16,17]. These findings support our hypothesis that HT may modulate the HSR by acting on CLU, which in turn activates the PI3K/Akt pathway.

Building on this research, our study investigates the effects of high temperature (HT) on CLU expression, the PI3K/Akt pathway, and the HSR in cardiomyocytes. We demonstrate that HT induces HSR through CLU upregulation via the PI3K/Akt pathway, thereby reducing apoptosis and protecting cells from HT-induced injury. These findings elucidate the molecular mechanisms underlying HT-triggered HSR and provide new insights into potential therapeutic strategies for myocarditis-related myocardial injury. For instance, regulating HSR by monitoring or modulating CLU expression may help mitigate the adverse effects of high fever.

## 2 Methods

### 2.1 Cell culture and treatment

**Figure 1:**
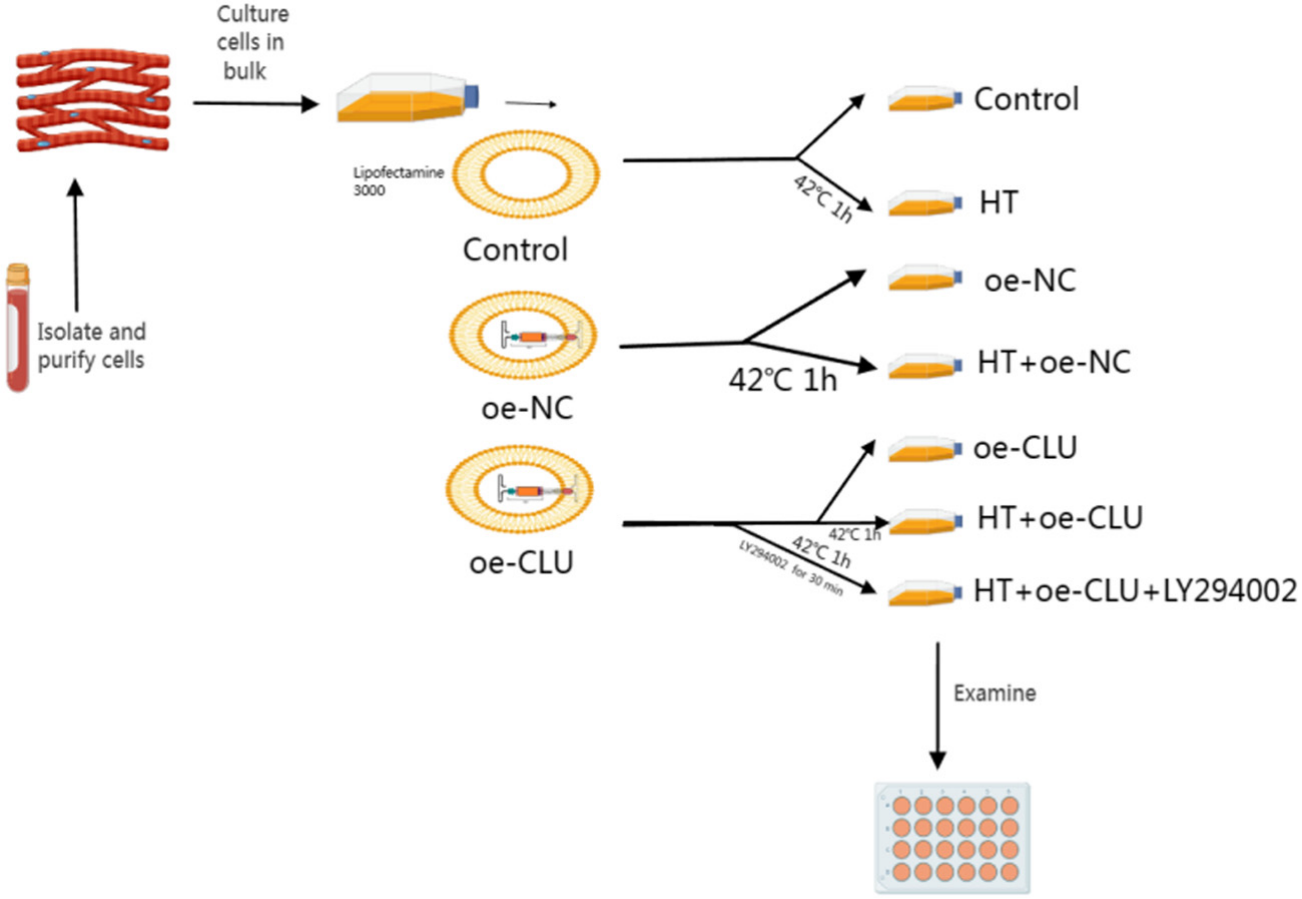
A schematic flowchart summarizing experimental groups and treatments.

Rat cardiomyocyte cell line (H9c2 cells) (CL-0089, Procell, Wuhan, China) was cultured in Dulbecco s modified eagle medium (21063029, Invitrogen, Waltham, USA) supplemented with 10% fetal bovine serum (A4766801, Invitrogen) and 100× penicillin–streptomycin solution (R01510, Invitrogen) at 37°C in a 5% CO₂ incubator. STR analysis confirmed cell authenticity and tested for mycoplasma contamination to ensure they were mycoplasma free. The cells were categorized into these experimental groups (Figure 1).

**Control:** Cardiomyocytes under normal conditions.

**HT:** Cells exposed to 42°C for 1 h and then returned to 37°C [18].

**oe-NC:** Normal cardiomyocytes transfected with the oe-NC vector.

**oe-CLU:** Normal cardiomyocytes transfected with the oe-CLU vector.

**HT + oe-NC:** Cardiomyocytes transfected with the oe-NC vector and subjected to HT exposure.

**HT + oe-CLU:** Cardiomyocytes transfected with the oe-CLU vector and subjected to HT exposure.

**HT + oe-CLU + LY294002:** Cardiomyocytes transfected with the oe-CLU vector, exposed to HT, and treated with the PI3K inhibitor LY294002 (10 μM) [19].

For cell transfection, the CDS sequence of CLU was cloned into the pGEM^®^-4Z Vector (P2161, Promega, Wisconsin, USA) to construct the CLU overexpression vector (oe-CLU). When cells reached 70–90% confluence and the cell count was approximately 5 × 10^5^, Lipofectamine 3000 (L3000015, Thermo Fisher Scientific, Waltham, USA) was used for transfection. A DNA solution was prepared by diluting 5 μg of plasmid DNA in 250 μL Opti-MEM, adding 10 μL of P3000™ Reagent, and mixing with a lipid solution made from 4 μL Lipo 3000 in 250 μL Opti-MEM. The DNA-lipid complex was added to the cells, and the cells were incubated at 37°C for 2 days before analyzing the transfected cells.

### 2.2 Cell counting kit-8 (CCK-8)

Cells were plated in a 96-well plate at 1 × 10^5^ cells per well and incubated at 37°C with 5% CO₂ for 0, 12, and 24 h. 10 μL of CCK-8 solution (HY-K0301, MedChemExpress, New Jersey, USA) was added to each well and the plate was incubated in the dark for 2 h. Absorbance was measured at 450 nm using a microplate reader (PerkinElmer, USA).

### 2.3 Reverse transcription quantitative polymerase chain reaction (RT-qPCR)

Total RNA was extracted using TRIzol reagent (15596026, Thermo Fisher Scientific) following the manufacturer’s instructions. RNA purification was then performed with the RNA Quick Purification Kit (61006, Thermo Fisher Scientific). Next, cDNA synthesis was carried out using the Reverse Transcription Kit (4368814, Thermo Fisher Scientific), and the cDNA was subsequently amplified with a PCR instrument. For quantitative PCR, SYBR Green PCR Master Mix (4368577, Thermo Fisher Scientific) was employed. The relative expression levels of target mRNA were normalized to the endogenous control GAPDH using the 2^−ΔΔCt^ method. The specific primer sequences utilized in this study are provided in Table 1.

**Table 1:** Primer sequences used for RT-qPCR (5′ to 3′)

Gene	Forward primer	Reverse primer
CLU	GACTCCAGACTCCAAAGAGGC	TGGACGGCGTTCTGAATCTC
HSP70	AGACAGACTCTTGATGGCTGC	TCGCAGGAAGGAAACACCAT
HSP90	GCTTGGTCTAGGTATTGATGAGGA	CCTTCCAGGGGTGGCATTTC
Bax	TGGCGATGAACTGGACAACA	GGAAAGGAGGCCATCCCAG
Bcl-2	GAACTGGGGGAGGATTGTGG	GGGGTGACATCTCCCTGTTG
GAPDH	CCGCATCTTCTTGTGCAGTG	ACCAGCTTCCCATTCTCAGC

Note: CLU = clusterin.

### 2.4 Western blot

Proteins were extracted from cells using radioimmunoprecipitation assay buffer (89901, Thermo Fisher Scientific). Protein concentration was measured using a BCA Protein Assay Kit (23225, Thermo Fisher Scientific) according to the manufacturer’s instructions. Equal amounts of protein (typically 20–30 µg per lane) were loaded onto sodium dodecyl sulfate polyacrylamide gel electrophoresis gels and run at 80 V for stacking and 120 V for separation, followed by transfer to a PVDF membrane at 100 V for 40–70 min. The membrane was blocked with 5% non-fat dry milk in tris-buffered saline with Tween (TBST) at 37°C for 1 h. After blocking, the membrane was incubated with primary antibodies at 25°C for 1 h: CLU (1:500, A13479, Abclonal, Wuhan, China), HSP70 (1:1,000, ab181606, Abcam, Cambridge, UK), HSP90 (1:10,000, ab203126, Abcam), Bax (1:1,000, ab32503, Abcam), Bcl-2 (1:500, ab194583, Abcam), Akt (1:1,000, 9272S, Cell Signaling Technology, Massachusetts, USA), p-Akt (1:2,000, 4060S, Cell Signaling Technology), PI3K (1:1,000, 4257S, Cell Signaling Technology), p-PI3K (1:1,000, SAB4504315, Sigma Aldrich, Missouri, USA), and GAPDH (1:10,000, ab181602, Abcam) and was used as the loading control. GAPDH was consistently used for the normalization of protein content. The membrane was then washed three times with TBST and incubated with secondary antibodies IgG H&L (HRP) (1:2,000, ab97051, Abcam), at 25°C for 1 h. Following three washes with TBST, chemiluminescent detection was performed using ECL reagent (P0018S, Beyotime, Shanghai, China), and protein levels were analyzed using ImageJ software (V1.8.0.112, NIH, Madison, WI, USA).

### 2.5 Flow cytometry

Cells were washed twice with cold phosphate-buffered saline (PBS), resuspended in 250 μL of binding buffer, and 100 μL of the suspension was transferred to a 5 mL flow cytometry tube. The experiment was performed according to the manufacturer’s instructions for the apoptosis detection kit. To the tube, 5 μL of Annexin V-FITC (A8604-200UL, Sigma-Aldrich) and 5 μL of PI (537059, Sigma-Aldrich) were added for dual staining. The staining was performed in the dark for 20 min. Analysis was performed using a flow cytometer (BD Biosciences, USA).

### 2.6 Intra-cellular reactive oxygen species (ROS) detection

1 × 10^6^ cells/mL were washed three times with PBS. Fresh medium was added, followed by a MitoSOX probe (S0061S, Beyotime) to a final concentration of 5 μM. The cells were incubated at 37°C in the dark for 30 min. After complete binding of the probe, the cells were washed with PBS to remove any residual probe. Immunofluorescence results were observed using a fluorescence microscope.

### 2.7 Statistical analysis

Data in the text were performed by technical replicates and were included alongside biological triplicates to strengthen reproducibility. Statistical analysis was performed using GraphPad Prism 9 (Dotmatics, Boston, MA, USA). The presented results, expressed as the mean ± standard deviation (SD) were obtained from three separate experiments, each carried out in triplicate. To assess statistical significance among groups, two groups were analyzed by *t*-test. One-way or two-way analysis of variance (ANOVA) followed by Tukey’s *post hoc* test was used for analyzing differences between three or more groups. The chosen significance threshold was *p* < 0.05.

## 3 Results

### 3.1 HT exposure induces cardiomyocyte apoptosis and activates the HSR

**Figure 2:**
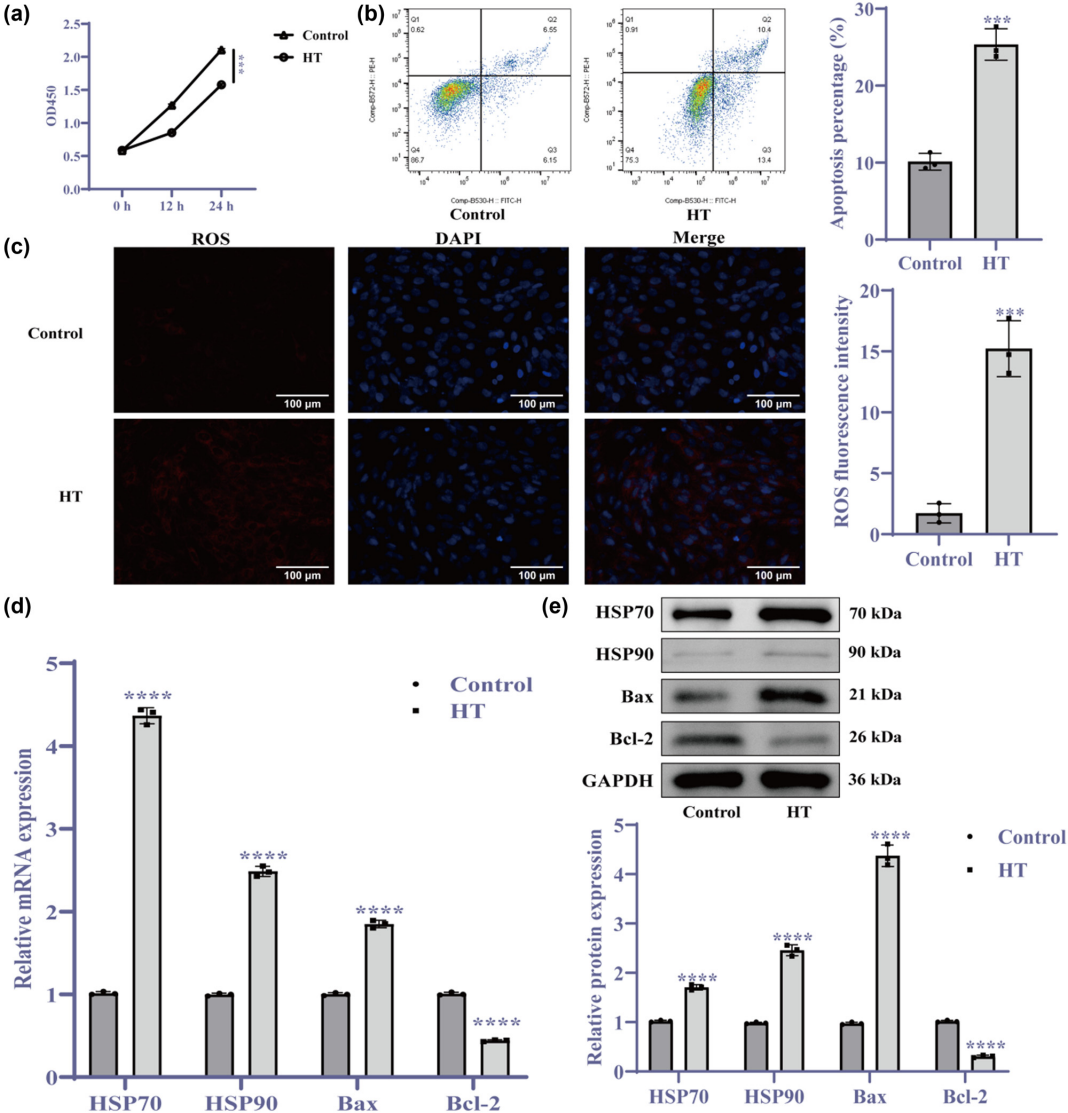
HT exposure induces cardiomyocyte apoptosis and activates the HSR. (a) CCK-8 assay assessing cell proliferation at 0, 12, and 24 h; (b) Flow cytometry for apoptosis quantification; (c) MitoSOX assay measuring mitochondrial ROS levels (400×); (d) RT-qPCR evaluating mRNA expression levels; and (e) Western blot analysis of protein expression levels. ***p < 0.001, ****p < 0.0001 vs control. *N* = 3. Abbreviations: HT = high temperature; CLU = clusterin.

To examine the effects of HT exposure on cardiomyocyte damage and HSR activation, we subjected cells to HT conditions and assessed the outcomes using various assays. The results revealed a significant reduction in cell proliferation in the HT group (Figure 2a, *p* < 0.001) along with a marked increase in apoptosis (Figure 2b, *p* < 0.001). Additionally, ROS levels were significantly elevated in the HT group (Figure 2c, *p* < 0.001). Protein expression analysis showed upregulation of Bax, downregulation of Bcl-2, and substantial increases in HSP70 and HSP90 levels (Figure 2d, *p* < 0.0001). Collectively, these findings indicate that HT exposure induces cardiomyocyte apoptosis and activates the HSR.

### 3.2 HT exposure upregulates CLU and CLU overexpression enhances HSR and reduces apoptosis

**Figure 3:**
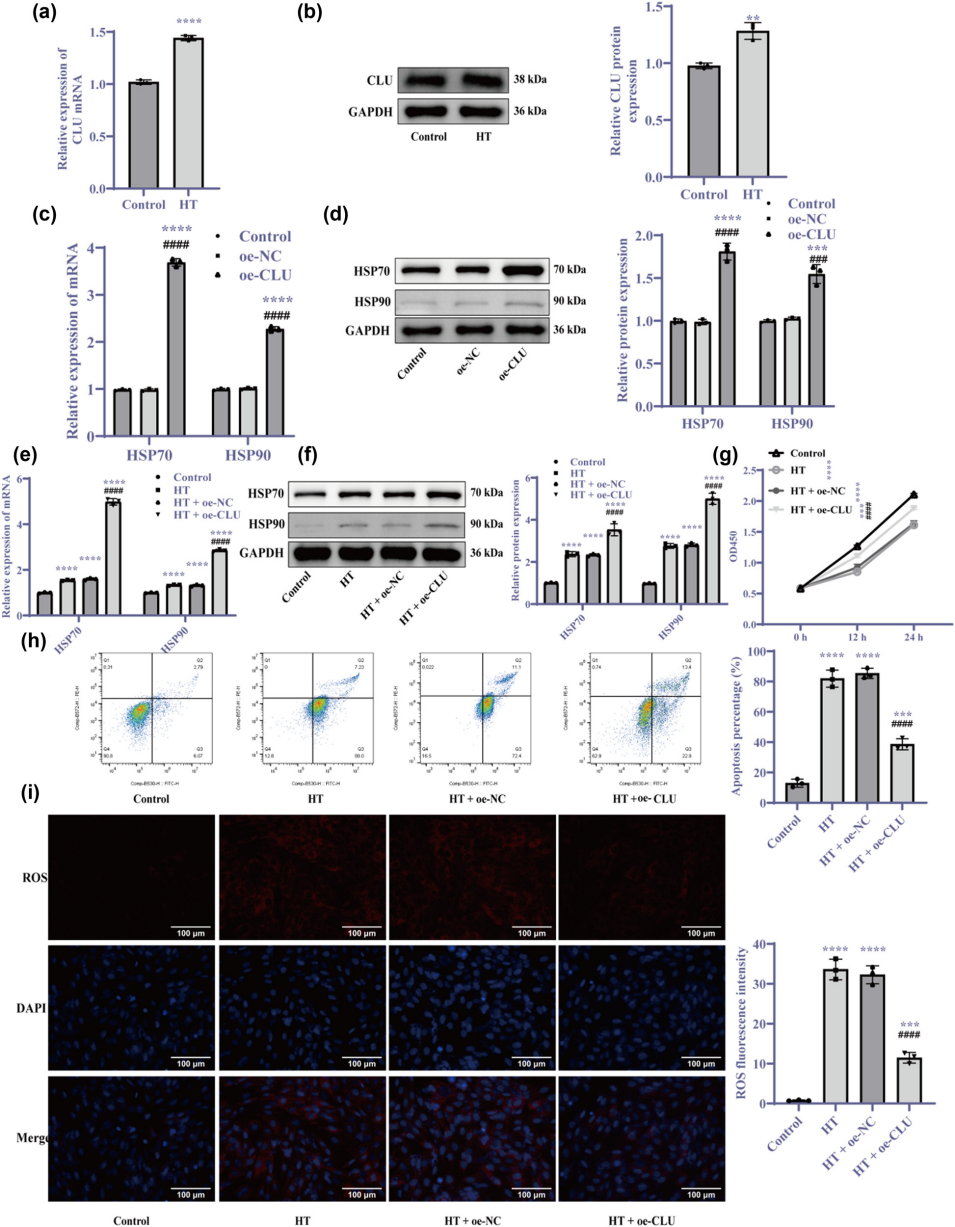
HT exposure upregulates CLU and CLU overexpression enhances HSR and reduces apoptosis. (a, c, and e) RT-qPCR analysis of mRNA expression levels; (b, d, and f) Western blot analysis of protein levels; (g) CCK-8 assay measuring cell proliferation; (h) Flow cytometry for apoptosis detection at 0, 12, and 24 h; and (i) MitoSOX assay assessing mitochondrial ROS levels (400×). Statistical analyses were performed using *t*-tests, one-way ANOVA, and two-way ANOVA followed by Tukey’s *post hoc* test. Results are presented as mean ± SD, with *N* = 3. Significant differences are indicated as follows: **p < 0.01, ***p < 0.001, ****p < 0.0001 vs control, ^###^ *p* < 0.001, ^####^ *p* < 0.0001 vs oe-NC or HT + oe-NC. Abbreviations: HT = high temperature; CLU = clusterin; NC = negative control.

Previous studies have shown that CLU can mediate the cellular HSR under HT conditions [20]. To assess the impact of HT exposure on CLU expression, we observed a significant upregulation of CLU in the HT group (Figure 3a and b, *p* < 0.01), indicating that HT exposure promotes CLU expression. To further investigate the relationship between CLU and HSR, and their effects on cellular ROS levels and proliferation, we transfected cells with a CLU overexpression vector. Compared to the HT + oe-NC group, the HT + oe-CLU group showed increased levels of CLU, HSP70, and HSP90, along with enhanced cell proliferation (Figure 3c and d, *p* < 0.001). These findings suggest that HT exposure not only upregulates CLU but also that CLU overexpression under HT conditions significantly increases the expression of HSP70 and HSP90. This activation of the HSR improves to improved cardiomyocyte proliferation while mitigating apoptosis and oxidative damage (Figure 3e–i, *p* < 0.001). Overall, CLU overexpression facilitates the expression of additional HSPs, which can partially reverse the cellular damage induced by HT exposure.

### 3.3 HT exposure inhibits PI3K/Akt signaling pathway activity

**Figure 4:**
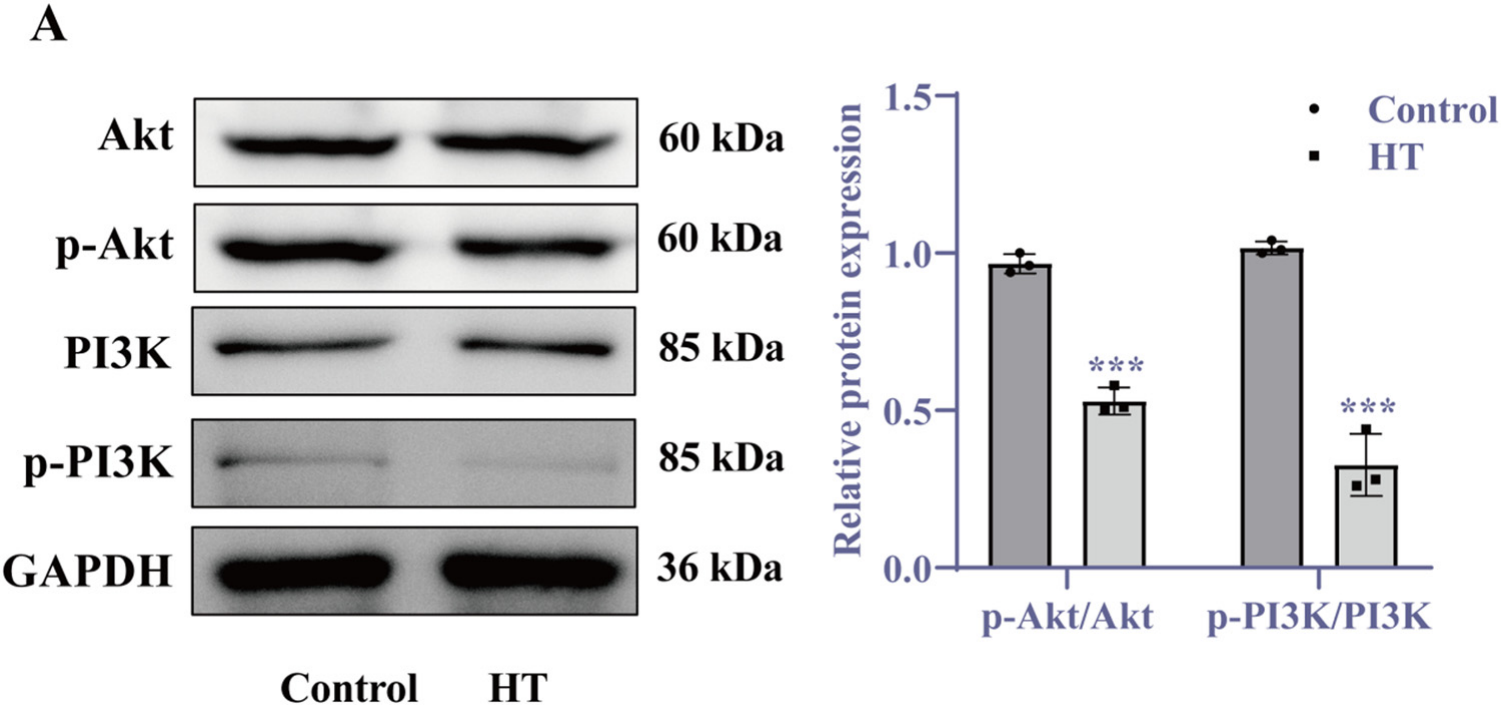
HT exposure inhibits PI3K/Akt signaling pathway activity. (a) Western blot analysis of protein levels along with quantification results. Statistical analyses were conducted using two-way ANOVA followed by Tukey’s *post hoc* test. Results are presented as mean ± SD, with *N* = 3. Significant differences are indicated as ***p < 0.001 vs control. Abbreviation: HT = high temperature.

Since HSP70 produced by HT induction alone is insufficient to counteract apoptosis, but CLU overexpression synergizes with HSP70 to inhibit apoptosis, we hypothesized the involvement of a molecular regulatory pathway. To explore this further, we analyzed the signaling pathways involved in temperature regulation and investigated the molecular mechanism of CLU under HT conditions. Previous studies have established a close relationship between temperature regulation and the PI3K/Akt signaling pathway [20]. To assess the impact of HT exposure on this pathway in cardiomyocytes, we evaluated the activation status of the PI3K/Akt signaling pathway under HT conditions. Our results indicated that the HT group exhibited a significant decrease in the ratios of p-PI3K to total PI3K and p-Akt to total Akt (Figure 4, *p* < 0.001). These findings suggest that HT exposure inhibits the activity of the PI3K/Akt signaling pathway in cardiomyocytes.

### 3.4 CLU overexpression activates PI3K/Akt signaling pathway and HSR

**Figure 5:**
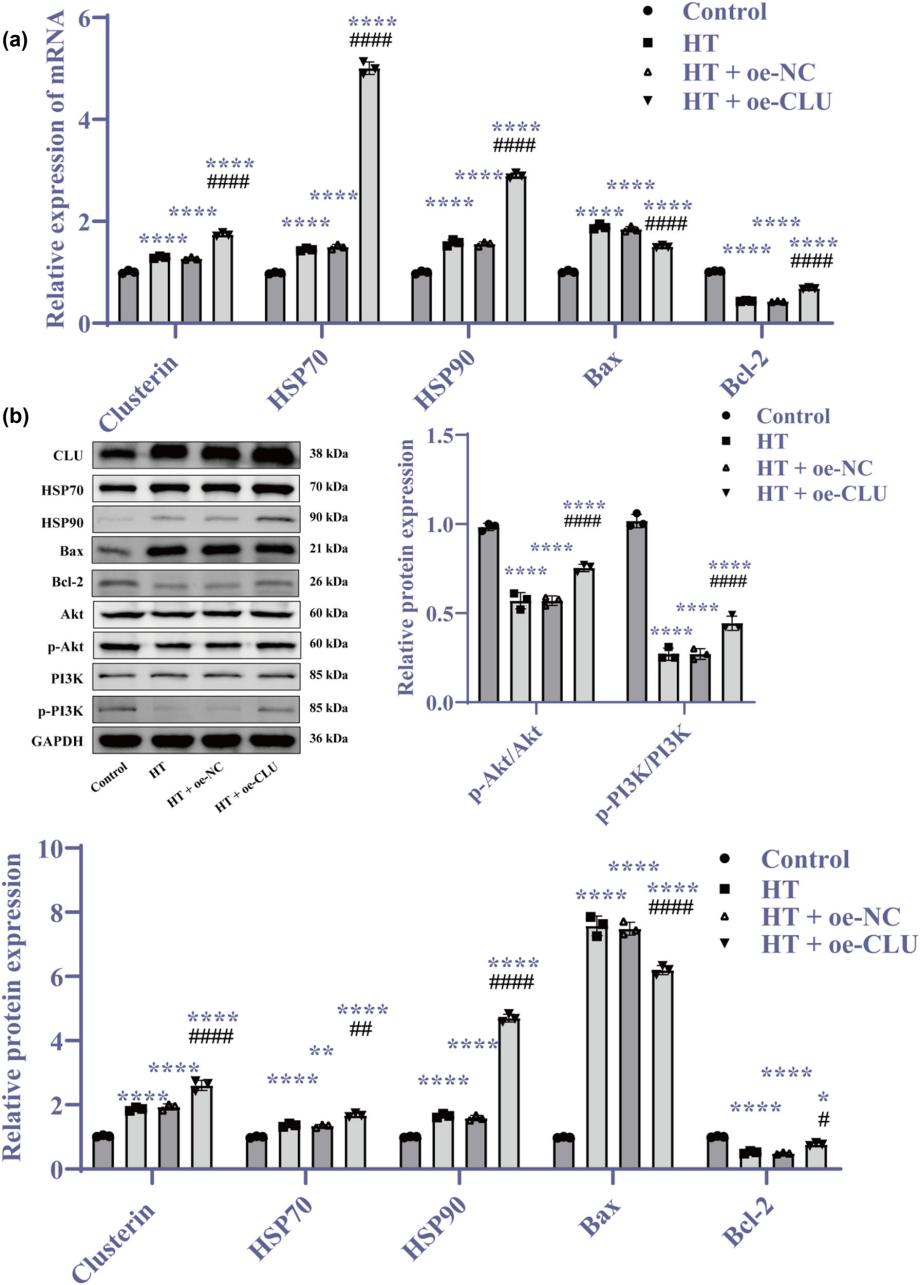
CLU overexpression activates PI3K/Akt signaling pathway and HSR. (a) RT-qPCR analysis of mRNA levels and (b) Western blot analysis of protein levels. Statistical analyses were performed using two-way ANOVA followed by Tukey’s *post hoc* test. Results are presented as mean ± SD, with *N* = 3. Significant differences are indicated as **p < 0.01, ****p < 0.0001 vs control, ^#^ *p* < 0.05, ^##^ *p* < 0.01, ^####^ *p* < 0.0001 vs HT + oe-NC. Abbreviations: HT = high temperature; CLU = clusterin; NC = negative control.

To further investigate the regulatory role of CLU on the PI3K/Akt signaling pathway, we assessed the effects of CLU overexpression in cardiomyocytes under HT conditions. Compared to the HT + oe-NC group, the HT + oe-CLU group exhibited increased levels of HSP70 and HSP90, a decrease in Bax, an increase in Bcl-2, and a significant elevation in the ratios of p-PI3K to total PI3K and p-Akt to total Akt (Figure 5a and b, *p* < 0.0001). These findings indicate that CLU overexpression activates the PI3K/Akt signaling and enhances the protective effects of HSR.

### 3.5 CLU overexpression activates the PI3K/Akt signaling pathway to promote HSR and alleviate cardiomyocyte apoptosis under HT conditions

**Figure 6:**
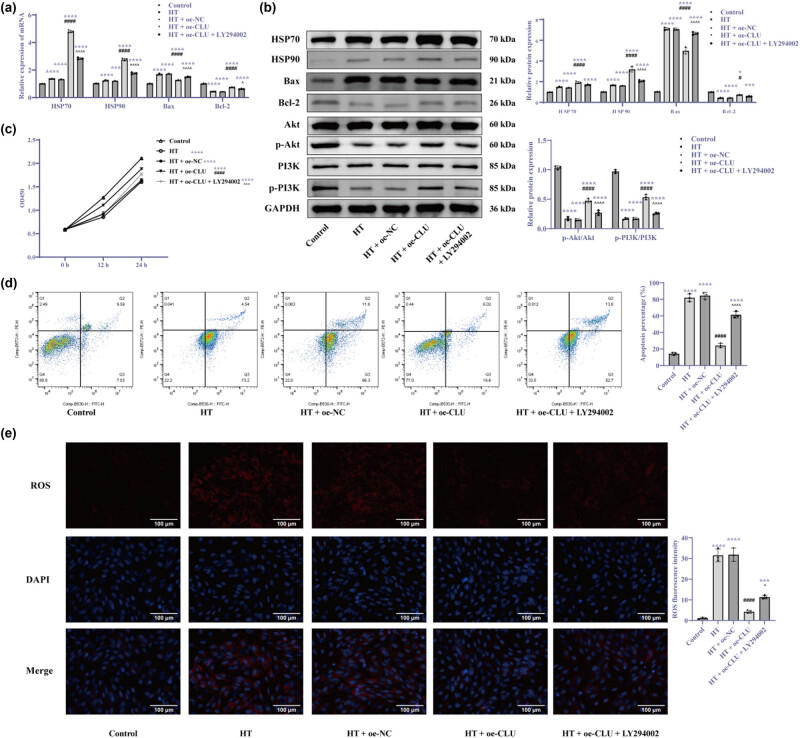
CLU overexpression activates the PI3K/Akt signaling pathway to promote HSR and alleviate cardiomyocyte apoptosis under HT conditions. (a) RT-qPCR analysis of mRNA levels; (b) Western blot analysis of protein levels; (c) CCK-8 assay for cell proliferation at 0, 12 and 24 h; (d) flow cytometry analysis of apoptosis; and (e) MitoSOX analysis of ROS levels (400×). Statistical analyses were performed using one-way ANOVA and two-way ANOVA followed by Tukey’s *post hoc* test. Results are presented as mean ± SD, with *N* = 3. Significant differences are indicated as *p < 0.05, ***p < 0.001, ****p < 0.0001 vs control, ^#^ *p* < 0.05, ^####^ *p* < 0.0001 vs HT + oe-NC, ^*p* < 0.05, ^^^*p* < 0.001, ^^^^*p* < 0.0001 vs HT + oe-CLU. Abbreviations: HT = high temperature; CLU = clusterin; NC = negative control.

To determine whether CLU activates the HSR through the PI3K/Akt pathway, we utilized LY294002 [21] to inhibit PI3K expression and then assessed the effects of CLU overexpression on cardiomyocytes. Compared to the HT + oe-CLU group, the HT + oe-CLU + LY294002 group exhibited a decrease in HSP70 and HSP90 levels, increased Bax, decreased Bcl-2, a significant reduction in the p-PI3K/total-PI3K and p-Akt/total-Akt ratios (Figure 6a and b, *p* < 0.01) Additionally, this group showed decreased cardiomyocyte proliferation (Figure 6c, *p* < 0.01), increased apoptosis (Figure 6d, *p* < 0.001), and elevated ROS levels (Figure 6e, *p* < 0.05). These results indicate that CLU overexpression under HT conditions promotes HSR by activating the PI3K/Akt signaling pathway, thereby enhancing cell proliferation and alleviating cardiomyocyte apoptosis.

## 4 Discussion

IM is characterized by inflammation of the myocardium due to infectious agents, with high fever being a common clinical manifestation [22]. This elevated body temperature typically results from the immune response triggered by the infection [23]. High fever can significantly compromise myocardial integrity by increasing the metabolic rate and oxygen demand of cardiomyocytes, ultimately leading to myocardial cell injury and dysfunction [24]. Additionally, the hyperthermic condition can intensify the inflammatory response, leading to further myocardial tissue damage [25]. The cardiovascular stress response induced by high fever may also increase the cardiac workload, exacerbating the condition [26]. Therefore, therapeutic interventions targeting high fever are crucial to mitigating its adverse effects on the myocardium, thereby improving patient recovery and prognosis.

The HSR is primarily characterized by the upregulation of HSPs, which act as molecular chaperones during periods of cellular stress, such as heat and oxidative stress [27]. These proteins help maintain proper protein folding and functionality, thereby preventing protein denaturation and aggregation. Notably, certain HSPs, including HSP27 and HSP70, also provide protective effects against apoptosis by modulating apoptotic pathways [28,29]. Interestingly, the present study elucidates that HT environments trigger cellular HSR, but the response is insufficient. This is consistent with the previous results [30,31].

In the context of cardiac-related diseases, heat shock has been shown to confer cardioprotective effects by modulating inflammation and regulating apoptosis [32,33]. For example, during myocardial ischemia-reperfusion injury – such as that occurring in cardiac surgery or myocardial infarction – the HSR helps attenuate inflammatory responses and reduce myocardial cell damage, ultimately limiting myocardial cell death [34]. Additionally, HSP70 and HSP27 protect cardiomyocytes from stress-induced apoptosis by inhibiting apoptotic pathways, further demonstrating their anti-apoptotic properties [34,35]. Our experimental findings corroborate previous reports, as we observed that HT upregulated the expression of HSP70 and HSP90; however, this response was insufficient to fully mitigate apoptosis induced by HT. In contrast, the overexpression of CLU significantly enhanced the expression of HSP70 and HSP90, while simultaneously inhibiting HT-induced apoptosis. These findings suggest that boosting the expression of HSPs could be a promising therapeutic strategy for managing heart diseases.

CLU, a highly conserved glycoprotein found in various tissues and body fluids [36,37], plays a crucial role in a wide range of biological processes, including cellular stress responses, apoptosis, inflammation, cytoprotection, and immune regulation [38]. The results of this study indicate that HT exposure upregulates CLU, which in turn activates the HSR and reduces myocardial apoptosis. Notably, CLU and HSPs exhibit numerous interactions and functional associations, both involved in essential processes such as protein folding, refolding, transport, and degradation, thus protecting cells from proteotoxic damage [39]. CLU and HSPs may act synergistically within the protein quality control system, especially under stress conditions [40]. Additionally, previous studies have shown that CLU can mitigate inflammatory responses by inhibiting the complement cascade and reducing the secretion of inflammatory mediators [41]. CLU has been found to attenuate ischemia-reperfusion injury associated with cardiac transplantation by modulating NF-kB signaling and regulating Bax/Bcl-xL expression [42]. Collectively, these findings highlight the potential of CLU in protecting cardiomyocytes from harmful stimuli. In line with our observations, CLU has also been reported to offer protective effects against stress-induced apoptosis [43].

Moreover, our investigation revealed that CLU overexpression in HT environments promotes the HSR through the activation of the PI3K/Akt signaling pathway, which coincides with the existing studies, this signaling cascade is integral to cellular survival, proliferation, and metabolic regulation [44,45]. PI3K is activated by various stimuli, including growth factors and insulin, resulting in the production of PIP3, which subsequently activates PDK1 and Akt [44]. Activated Akt further modulates multiple downstream effectors involved in the regulation of cell survival and apoptosis [46]. Notably, prior research has indicated that CLU can influence the expression of PI3K [47] and inhibit cellular senescence via the PI3K/Akt pathway [48]. Additionally, Yang et al. found that CLU can activate this pathway to reduce apoptosis [49]. However, this study is the first to demonstrate that HT influences apoptosis by regulating CLU to activate the PI3K/Akt pathway. However, this study lacks the support of *in vivo* validation and clinical data, which will be the focus of our further research.

## 5 Conclusion

In conclusion, our findings indicate that overexpression of CLU can trigger the HSR by activating the PI3K/Akt pathway, thereby affording protection to cardiomyocytes from HT-induced apoptosis and inflammatory responses. These insights may inform the development of novel therapeutic strategies targeting CLU and the HSR in the clinical management of myocardial injury associated with high fever. It also provides some data support for the potential development of gene therapy approaches or small-molecule modulators in CLU. Our future research direction will focus on examining the role of CLU in different cell types and further exploring its interaction with other stress-related pathways.

## Supplementary Material

Supplementary Figure
