# Supplementary figures and images for: Clusterin activates the heat shock response via the PI3K/Akt pathway to protect cardiomyocytes from high-temperature-induced apoptosis
Source: Open Life Sci. 2025 Mar 28;20(1):20251082. doi: 10.1515/biol-2025-1082 (PMC11964181; doi:10.1515/biol-2025-1082)

# Supplementary material

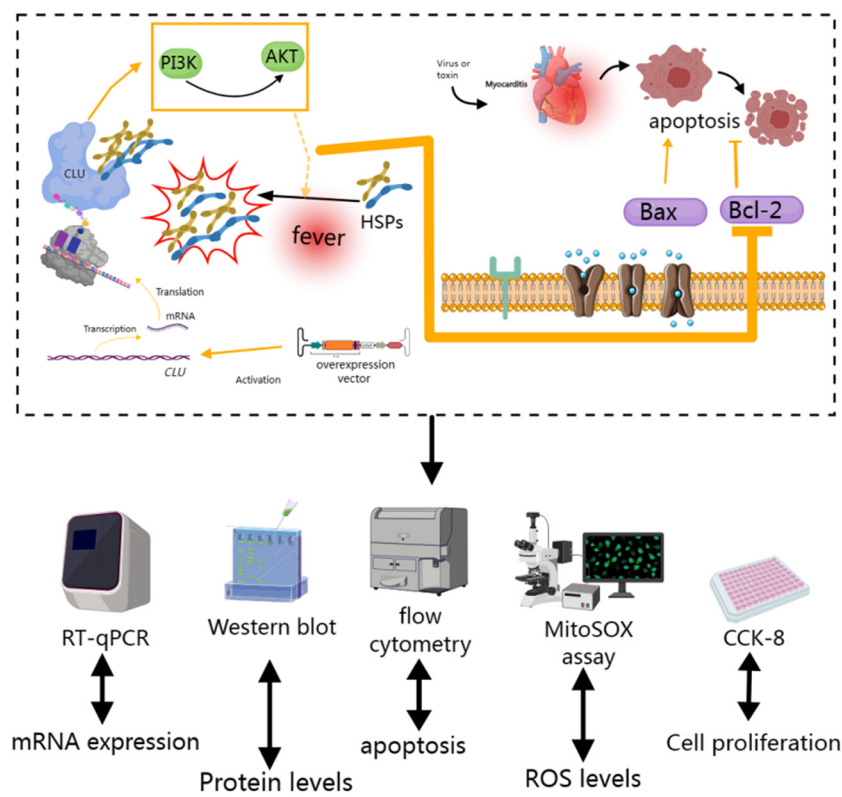

**Figure S1:** The mechanistic diagram of this study.

Supplement: Supplementary Figure [file biol-2025-1082-sm.pdf]
